# Modulation of Mcl-1 sensitizes glioblastoma to TRAIL-induced apoptosis

**DOI:** 10.1007/s10495-013-0935-2

**Published:** 2013-11-09

**Authors:** Á. C. Murphy, B. Weyhenmeyer, J. Noonan, S. M. Kilbride, S. Schimansky, K. P. Loh, D. Kögel, A. G. Letai, J. H. M. Prehn, B. M. Murphy

**Affiliations:** 1 Centre for Systems Medicine, Department of Physiology and Medical Physics, Royal College of Surgeons in Ireland, York House, St. Stephen’s Green, Dublin, 2 Ireland; 2 Experimental Neurosurgery, Centre for Neurology and Neurosurgery, Johann Wolfgang Goethe University Hospital, Theodor-Stern-Kai 7, 60590 Frankfurt, Germany; 3 Department of Biological Chemistry and Molecular Pharmacology, Harvard Medical School, Boston, MA 02115 USA; 4 Department of Medical Oncology, Dana-Farber Cancer Institute, Boston, MA 02215 USA

**Keywords:** Glioblastoma, TRAIL, R-roscovitine, Mcl-1

## Abstract

Glioblastoma (GBM) is the most aggressive form of primary brain tumour, with dismal patient outcome. Treatment failure is associated with intrinsic or acquired apoptosis resistance and the presence of a highly tumourigenic subpopulation of cancer cells called GBM stem cells. Tumour necrosis factor-related apoptosis-inducing ligand (TRAIL) has emerged as a promising novel therapy for some treatment-resistant tumours but unfortunately GBM can be completely resistant to TRAIL monotherapy. In this study, we identified Mcl-1, an anti-apoptotic Bcl-2 family member, as a critical player involved in determining the sensitivity of GBM to TRAIL-induced apoptosis. Effective targeting of Mcl-1 in TRAIL resistant GBM cells, either by gene silencing technology or by treatment with R-roscovitine, a cyclin-dependent kinase inhibitor that targets Mcl-1, was demonstrated to augment sensitivity to TRAIL, both within GBM cells grown as monolayers and in a 3D tumour model. Finally, we highlight that two separate pathways are activated during the apoptotic death of GBM cells treated with a combination of TRAIL and R-roscovitine, one which leads to caspase-8 and caspase-3 activation and a second pathway, involving a Mcl-1:Noxa axis. In conclusion, our study demonstrates that R-roscovitine in combination with TRAIL presents a promising novel strategy to trigger cell death pathways in glioblastoma.

**Electronic supplementary material:**

The online version of this article (doi:10.1007/s10495-013-0935-2) contains supplementary material, which is available to authorized users.

## Introduction

Glioblastoma (GBM) is the most common and aggressive primary brain tumour that occurs in humans. The current standard of care for GBM patients is surgical resection followed by chemotherapeutic treatment with the alkylating cytostatic drug temozolomide, in combination with radiotherapy [1]. Despite this treatment strategy, the average period of survival of GBM patients is approximately 12 months [2].

GBM cells display extreme resistance to apoptotic stimuli which contributes to the limited effectiveness of current therapies and the difficulty in developing new efficacious treatment regimes. Contributing to this phenomenon in GBM is the overexpression of numerous survival proteins and attenuated levels of several pro-apoptotic proteins [3–5] which results in resistance to the execution of both intrinsic and extrinsic apoptotic pathways. Apoptotic resistance in GBM is compounded by the presence of a highly tumourigenic subpopulation of cancer cells called GBM stem cells (GSCs) [6, 7] that are extremely chemoresistant and radioresistant and drive the growth and progression of the tumour, especially after treatment [8–12]. Therefore, the resistance to apoptotic stimuli of both the differentiated tumour cells and the GSCs must be actively targeted by GBM therapy to ensure complete eradication of the tumour [8, 11].

A promising novel therapy for treatment-resistant tumours has emerged in recent years in the form of the extracellular ligand, tumour necrosis factor-related apoptosis-inducing ligand (TRAIL). TRAIL induces apoptosis when it binds the death receptors DR4/TRAIL-R1 and DR5/TRAIL-R2, leading to DISC formation and the processing and activation of procaspase-8 [13–15]. The apoptotic signal from the DISC may be inhibited by cellular FLICE-inhibitory protein (FLIP) [16]. However, once procaspase-8 has been activated, TRAIL-induced apoptotic signalling may be propagated either by a mitochondrial-independent or a mitochondrial-dependent pathway [14]. TRAIL was originally identified as an attractive candidate for clinical use as it selectively induces apoptosis in cancer cells while sparing normal tissue [14, 17]. Indeed such cancer-selective properties of TRAIL have been identified for glioma cells in comparison to non-neoplastic astrocytes in vitro [18]. Unfortunately, numerous studies have demonstrated that many malignancies, including GBM, are completely resistant to monotherapy with TRAIL [17, 19].

This resistance may be due to the overexpression of anti-apoptotic Bcl-2-like proteins, such as Bcl-2 and Bcl-x_L_ [20–22]. Many reports have also suggested that sensitivity to TRAIL-induced apoptosis is dependent on the expression levels of another anti-apoptotic Bcl-2 family member, myeloid cell leukemia-1 (Mcl-1) [23–25]. Mcl-1 is primarily localized at the mitochondrial membrane [26] where it can block the release of cytochrome *c* [27] thus inhibiting apoptosis. Like other anti-apoptotic Bcl-2 family members, Mcl-1 interacts with high affinity to the pro-apoptotic BH3-only proteins Bim, Bid and Puma; but it also selectively interacts with Noxa and Bak, blocking their pro-apoptotic function [28–30]. The contribution of Mcl-1 to TRAIL resistance in GBM is currently unknown.

In this study, we examine the role of Mcl-1 in the resistance of GBM to TRAIL and identify R-roscovitine as a potential candidate drug that targets Mcl-1 to enhance the therapeutic effects of TRAIL in the treatment of TRAIL resistant GBM cells and in a 3D tumour model.

## Materials and methods

### Cell culture of GBM cell lines and neurospheres

For this study, glioblastoma cell lines A172 and U87 and a glioblastoma cell line derived from a primary tumour, MZ-294 [22], were grown as a monolayer in DMEM with 10 % heat-inactivated fetal calf serum, 100 U/ml penicillin, and 100 mg/ml streptomycin and maintained in a humidified incubator at 37 °C and 5 % CO_2_. To generate MZ-294 neurospheres, the MZ-294 monolayer cells were washed in PBS (5×) and 1 × 10^6^ cells were seeded in a T25 flask (Greiner) or 2 × 10^5^ cells seeded in a six well-plate in DMEM-F12 medium (Gibco) containing 5 % sodium bicarbonate buffer solution (Life technologies), B27 supplement (Life technologies), penicillin (100 U/ml, Sigma), streptomycin (100 mg/ml, Sigma), Hepes buffer (1 M; Gibco), ITS liquid media supplement (Sigma), d-glucose solution (45 %; Sigma), progesterone (20 μM, Sigma), putrescine (100 μM, Sigma), heparin (2 %, Sigma), epidermal growth factor (0.1 μg/ml; Peprotech) and fibroblast growth factor (0.01 μg/ml; Peprotech) and maintained in a humidified incubator at 37 °C and 5 % CO_2_. The neurospheres were visualised using an Eclipse TE 300 inverted microscope (Nikon, Düsseldorf, Germany).

### MTT cell viability assay

MZ-294, U87 and A172 cells were plated in 96-well plates (2,000 cells/well) and treated with R-roscovitine (20 μM; Cayman Chemical) or TRAIL (100 ng/ml; Enzo Life Science) for 24, 48 and 72 h. In addition, MZ-294 were plated in 96-well plates (2,000 cells/well) and treated with R-roscovitine (20 μM), TRAIL (100 ng/ml), R-roscovitine (20 μM) + TRAIL (100 ng/ml) for 48 h. Following treatment, thiazolyl blue tetrazolium bromide (MTT, 5 mg/ml; Sigma) was added to each well and incubated in the dark at 37 °C. MTT produces a yellowish solution that is converted to dark blue, water-insoluble MTT formazan by mitochondrial dehydrogenases of living cells. After 4 h, the medium was aspirated and the dark blue crystals were dissolved in DMSO (200 μl). The absorbance of each sample was measured at 560 nm using a microplate reader (GENios, Tecan). The absorbance was proportional to the number of viable cells and expressed relative to control treated cultures.

### Transfection of GBM cell lines

A172, U87 and MZ-294 cells were transfected with human Mcl-1 knockdown 29mer shRNA construct in a retroviral GFP vector (Origene) and MZ-294 cells were transfected with human Noxa knockdown 29mer shRNA construct in a retroviral GFP vector (Origene) using calcium phosphate transfection kit (Life Technologies) as per manufacturer’s instructions.

### Hoechst staining of nuclear chromatin

Cells were treated as indicated and stained with Hoechst 33258 (1 μg/ml; Sigma), incubated for 10 min at 37 °C and nuclear morphology was visualised using an Eclipse TE 300 inverted microscope (Nikon, Düsseldorf, Germany) using a 20 × dry objective. A minimum of 300 cells were counted in three subfields of each culture. Those cells with condensed/fragmented nuclei were deemed apoptotic, counted using Image J software and expressed as a percentage of total cell number. In the case of transfected cells, only GFP^+^ cells were counted and apoptotic cells were expressed as a percentage of GFP^+^ cells.

### Western blot analysis

Western blot analysis was performed on lysates prepared from MZ-294, A172 and U87 cells treated as indicated. Cells were homogenised in lysis buffer containing 0.5 mmol/l Tris-Cl (pH 6.8), 2 % SDS (w/v), 10 % glycerin (w/v) and protease and phosphatase inhibitor cocktails (Sigma-Aldrich, Dublin). After determining the protein concentration of the samples using a BCA protein assay (Pierce, Illinois, USA), 20 μg samples were boiled in gel-loading buffer and separated on 10–15 % SDS-PAGE gels. Proteins were transferred to nitrocellulose membranes using the iBlot^®^ gel transfer device (Life Technologies, Ireland). The membranes were incubated with anti-human Mcl-1 (BD Biosciences, Oxford, UK), anti-human procaspase-8 and cleaved caspase-8, anti-human Bid and tBid, anti-human procaspase-3 and cleaved caspase-3 antibodies (Cell signalling Technology, Beverly, MA, USA) and anti-human Noxa, anti-human DR4 and anti-human DR5 (Abcam, Cambridge, UK) overnight at 4 °C. Membranes were next incubated with horseradish peroxidase-conjugated secondary antibodies (Jackson ImmunoResearch, Plymouth, PA, USA) and protein bands were visualised using Supersignal West Pico Chemiluminescent Substrate (Pierce, Rockford, IL, USA). Images were captured using Fuji-film LAS-3000 (Fuji, Sheffield, UK).

### Flow cytometry

MZ-294, A172 and U87 cells were plated in 24-well plates (15,000 cells/well) and treated with R-roscovitine (20 μM) for 24, 48 and 72 h. MZ-294 neurospheres and MZ-294 monolayer cells were treated with R-roscovitine (20 μM), TRAIL (100 ng/ml) and R-roscovitine (20 μM) + TRAIL (100 ng/ml) for 48 h. Following treatment, monolayer cells were harvested with trypsin–EDTA and washed with PBS. In the case of neurospheres, spheres were pelleted, dissociated in accutase (Life Technologies) for 10 min at 37 °C and washed in PBS. Cells were then incubated at room temperature in binding buffer (10 mM HEPES, 135 mM NaCl, 5 mM CaCl_2_) which contained an Annexin V-FITC conjugate (1 μl/ml; BioVision, Mountain View, CA, USA) and propidium iodide (PI; 1 μl/ml, BioVision) for 15 min. Cells were counted in a Cyflow ML 16 flow cytometer (Partec, Münster, Germany). Excitation of Annexin V-FITC was done with a 488 nm laser and fluorescence emission was collected in the FL1 channel through a 520 nm band pass filter. PI was excited with a 488 nm laser and fluorescence emission was collected in the FL3 channel through a 620 nm long pass filter. 1 × 10^4^ gated cells were acquired for each sample and analyzed using the Flowmax software (Partec).

### Measurement of Noxa-induced MOMP in MZ-294 and A172 cells

Measurement of mitochondrial depolarisation, as indicated by ratiometric fluorescent dye JC1, was assessed in A172 and MZ-294 cells treated with Noxa BH3 peptide as previously described [31]. Noxa BH3 peptide (synthesised by Tufts University Core Facility), a negative control (DMSO) and a positive control carbonyl cyanide 4-(trifluoromethoxy)-phenylhydrazone (FCCP) were deposited into each well in triplicate in T-EB (15 μl; 300 mM Trehalose, 10 mM Hepes-KOH pH 7.7, 80 mM KCl, 1 mM EGTA, 0.1 % BSA, 5 mM succinate) in a black 384-well plate (BD Falcon). Single cell suspensions of MZ-294 and A172 cells were washed in T-EB before being resuspended at 4 × their final density (1 × 10^4^ cells/well). One volume of the 4 × cell suspension was added to one volume of a 4 × dye solution containing 4 μM JC-1 (Life technologies), 40 μg/ml oligomycin, 0.02 % digitonin, 20 mM 2-mercaptoethanol in T-EB. This 2 × cell/dye solution was incubated for 5–10 min at RT to allow permeabilization and dye equilibration. A total of 15 μl of the 2 × cell/dye mix was then added to each treatment well to give a final Noxa BH3 peptide concentration of 100 μM. The plate was shaken for 15 s inside the plate reader (Tecan Safire 2), and the fluorescence at 590 nm was recorded every 5 min at RT for 3 h. The area under the curve of JC-1 fluorescence intensity (545 nm excitation and 590 nm emission) was calculated, and the depolarization reported in response to Noxa BH3 peptide is normalized relative to the area under the curve in the presence of a negative control, DMSO (0 %), and a positive control, the mitochondrial uncoupling agent FCCP (100 %).

### Immunostaining

For immunocytochemical staining, MZ-294 monolayer cells were grown on 13 mM coverslips (Fisher Scientific Ireland) in 24 well plates (15,000 cells/well). MZ-294 neurospheres were dissociated in accutase at 37 °C for 10 min and the single cell suspension (3 ×  10^5^ cells/ml) was cytospun onto glass slides (VWR International) using a Shandon CytoSpin III Cytocentrifuge as per manufacturer’s instructions. Cells on the coverslips and slides were fixed in ice cold methanol for 10 min, blocked in 5 % goat serum, washed in PBS containing 0.2 % Tween (4×) and incubated overnight with rabbit anti-human CD133 antibody (Cell signalling Technology, Beverly, MA, USA) at 4 °C. Cells were washed in PBS containing 0.2 % Tween (4×) and incubated with PE-conjugated secondary antibodies (Life technologies, Molecular Probes). Cells were then incubated overnight with mouse anti-human nestin antibody (R and D Systems) at 4 °C, washed in PBS containing 0.2 % Tween (4×) and incubated with FITC-conjugated secondary antibodies (Life technologies, Molecular Probes). Lastly, coverslips were mounted on glass slides and glass slides were covered with rectangular coverslips using DAPI-containing VectaShield (Vector Laboratories, CA). Staining was visualised using an Eclipse TE 300 inverted microscope (Nikon, Düsseldorf, Germany) using a 20× dry objective.

### Gene expression analysis using quantitative real-time RT-PCR

Total RNA was extracted using the RNeasy Mini Kit (Qiagen, UK). First-strand cDNA synthesis was performed with 1 μg total RNA as template and Superscript II reverse transcriptase (Life technologies, UK) primed with 50 pmol random hexamers (Thermo Scientific, USA). Quantitative real-time PCR was performed using a LightCycler 1.5 (Roche Diagnostics, UK) in combination with QuantiTect^®^ SYBR^®^ Green PCR kit (Qiagen) as per manufacturer’s protocol. The data were analysed using LightCycler Software 4.0^®^, normalized to expression of β-actin and represented as relative quantification values. Specific primers were designed using Primer3 software (http://frodo.wi.mit.edu/primer3/). Sense and antisense primer sequences were as follows Mcl-1 Forward: TGC TGG AGT AGG AGC TGG TT, Mcl-1 Reverse: CCT CTT GCC ACT TGC TTT TC, Noxa Forward: AGT CGA GTG TGC TAC TCA ACT A, Noxa Reverse: TGA TGT ATT CCA TCT TCC GTT T.

### Statistical analysis

Statistical analysis of the data was carried out on GraphPad InStat software using *t* tests, one-way ANOVA followed by Student–Newman–Keuls post hoc test and two-way ANOVA followed by Bonferroni post hoc test, where appropriate. When the *p* value was <0.05, groups were considered to be significantly different.

## Results

### TRAIL resistance in GBM cell lines can be attenuated by decreasing expression levels of Mcl-1

**Fig. 1:**
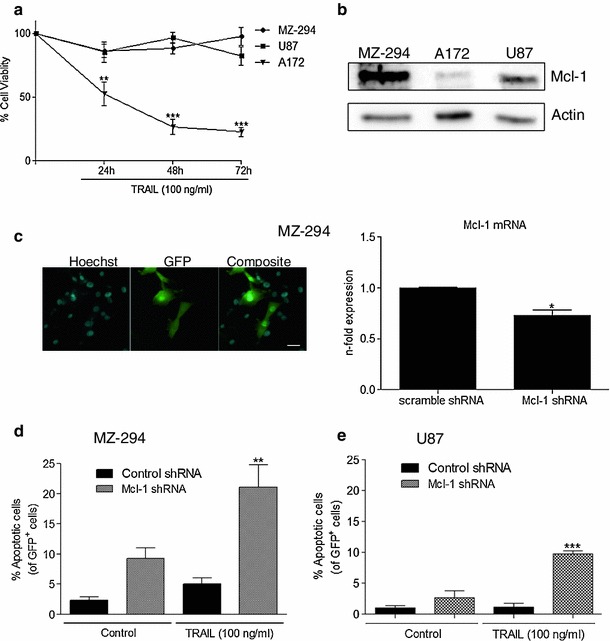
Mcl-1 downregulation enhances TRAIL-induced apoptosis in MZ-294 and U87 GBM cell lines. **a** Commercially available GBM cell lines A172 and U87, and a cell line derived from a primary tumour, MZ-294, were treated with TRAIL (100 ng/ml) for 24, 48 and 72 h. After treatment, MTT assays were carried out to assess cell viability. Data are expressed as mean ± SEM, **p < 0.01; ***p < 0.001 versus MZ-294 cells at the same timepoint, three independent experiments. **b** Basal levels of Mcl-1 were assessed in MZ-294, A172 and U87 cells by western blotting. Actin was used as a loading control. **c** Images (*scale bar* = 20 μM) depicting transfection efficiency of the GFP-expressing scramble control and Mcl-1 knockdown shRNA in MZ-294 cells and the subsequent knockdown of Mcl-1 after 24 h are shown. **d** Following transfection with a GFP-expressing Mcl-1 knockdown shRNA and treatment with TRAIL (100 ng/ml) for 48 h, TRAIL-induced apoptosis of GFP^+^ cells was assessed by Hoechst staining in MZ-294 cells. **e** TRAIL-induced apoptosis of GFP^+^ cells was also assessed in U87 cells by Hoechst staining. Data are expressed as mean ± SEM, **p < 0.01; ***p < 0.001 versus TRAIL only treated cells. Data are from three independent experiments

In order to investigate TRAIL sensitivity and resistance in GBM, a panel of three GBM cell lines, consisting of two commercially available lines, A172 and U87 and a cell line derived from a primary GBM tumour, MZ-294, were treated with TRAIL for 24, 48 and 72 h (Fig 1a). All of these cell lines have previously been shown to express the TRAIL receptor DR5/TRAIL-R2 [22]. Following treatment, U87 and MZ-294 cells were found to be resistant to TRAIL-induced effects on cell viability. However, the percentage of viable A172 cells was significantly decreased following TRAIL treatment at all timepoints (Fig 1a). Given that previous studies have linked Mcl-1 expression levels and sensitivity to TRAIL treatment, we investigated the basal levels of Mcl-1 expression in the cell lines and observed that the TRAIL resistant cell lines, U87 and MZ-294, expressed higher Mcl-1 protein levels in comparison to the TRAIL sensitive cell line, A172 (Fig 1b). We therefore next investigated the effect of suppression of Mcl-1 expression using gene silencing technology on TRAIL-induced apoptosis in the treatment-resistant MZ-294 and U87 cells (Fig 1d, e). The cells were transfected with a GFP vector expressing a Mcl-1 targeting shRNA construct (Fig 1c; left panel) and the resultant reduction in Mcl-1 expression was quantified using qPCR (Fig 1c; right panel). The cells were subsequently treated with TRAIL for 48 h and counting transfected cells only, we observed that the cells in which Mcl-1 expression was downregulated, were significantly more sensitive to TRAIL-induced apoptosis (Fig 1d, e).

### Expression levels of Mcl-1 and Noxa in GBM cell lines determine their resistance to R-roscovitine-induced apoptosis

**Fig. 2:**
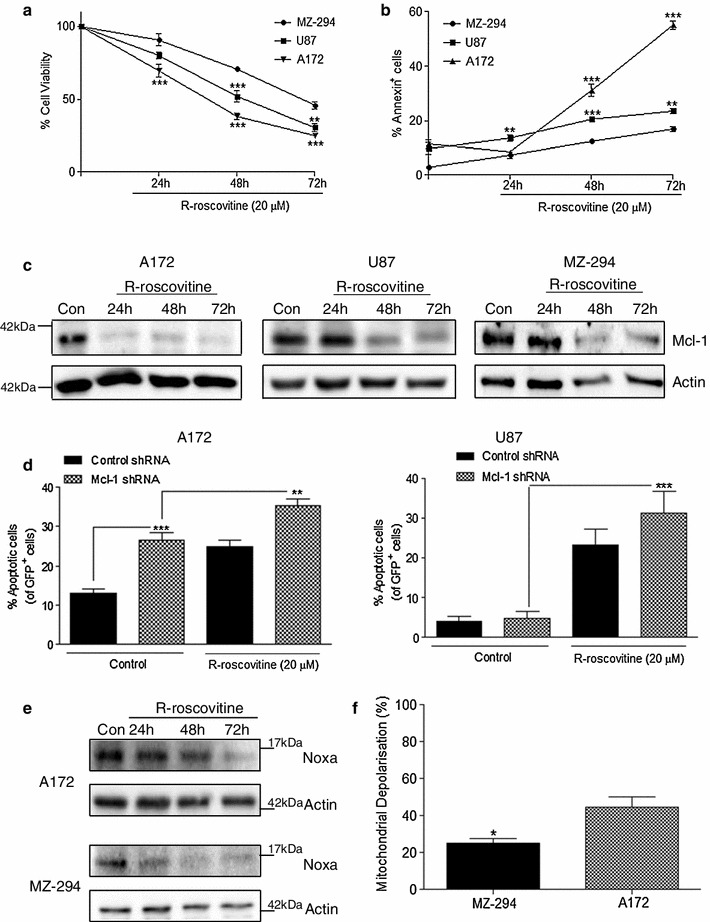
Knockdown of Mcl-1 augments R-roscovitine-induced apoptosis in A172 and U87 GBM cell lines. The GBM cells lines A172, U87 and MZ-294 were treated with R-roscovitine (20 μM) for 24, 48 and 72 h. **a** Cell viability was assessed using MTT assay and **b** the induction of apoptosis was determined by Annexin staining using flow cytometry. Data are expressed as mean ± SEM, **p < 0.01; ***p < 0.001 versus MZ-294 cells at the same timepoint. Data are from three independent experiments. **c** Western blot analysis of Mcl-1 expression was carried out on A172, U87 and MZ-294 cells treated with R-roscovitine (20 μM) for 24, 48 and 72 h. Actin was used as a loading control. **d** A172 and U87 cells were transfected with GFP-expressing Mcl-1 knockdown shRNA and subsequently treated with R-roscovitine (20 μM) for 48 h. The percentage of apoptotic GFP^+^ cells was determined by Hoechst staining. Data are expressed as mean ± SEM, **p < 0.01; ***p < 0.001; data are from three independent experiments. **e** A172 and MZ-294 cells were treated with R-roscovitine (20 μM) for 24, 48 and 72 h and the expression of Noxa was determined by western blot analysis. Actin was used as a loading control. **f** Mitochondrial depolarisation, indicated by the ratiometric fluorescent dye JC1, was measured in A172 and MZ-294 cells treated with Noxa BH3 peptide (100 μM). Depolarisation with Noxa BH3 peptide was expressed as a percentage of depolarisation induced by the protonophore, FCCP (100 %) compared to vehicle (DMSO), *p < 0.05; data are from three independent experiments

Since the attenuation of Mcl-1 expression partially re-sensitized the resistant cell lines to TRAIL-induced apoptosis, we next examined the effect of treatment with R-roscovitine, a known downregulator of Mcl-1, in our panel of cell lines [32]. The cells were treated with R-roscovitine for 24, 48 and 72 h. Treatment with R-roscovitine significantly decreased cell survival of the A172 and U87 cell lines compared with the MZ-294 cell line (Fig 2a) and significantly increased the percentage of these cells undergoing apoptosis compared with the MZ-294 cells (Fig 2b). Next we assessed the consequences of R-roscovitine treatment on Mcl-1 expression and found that Mcl-1 expression was downregulated at the protein level in A172 cells as early as 24 h post treatment (Fig 2c) and while R-roscovitine did decrease Mcl-1 expression in U87 (Fig 2c), it did so to a lesser extent and at a later timepoint than in A172 cells, corresponding to the lower levels of cell death evident in this cell line (Fig 2b). The sole targeting of Mcl-1 expression in A172 cells using shRNA technology significantly increased the percentage of cells undergoing apoptosis (Fig 2d) while downregulation of Mcl-1 significantly potentiated the induction of cell death with subsequent R-roscovitine treatment in both A172 and U87 cells (Fig 2d).

We also observed that treatment of MZ-294 cells with R-roscovitine decreased Mcl-1 levels in this cell line (Fig 2c), without causing significant levels of cell death (Fig 2b). As Noxa has been shown to be a critical effector of apoptosis due to its ability to neutralize Mcl-1 [25, 33], we next assessed the expression levels of Noxa during the apoptotic death of the cells. Following R-roscovitine treatment, we observed that expression of Noxa was targeted in both A172 and MZ-294 cells (Fig 2e). Interestingly however, using BH3 profiling we observed that A172 cells underwent greater levels of mitochondrial depolarisation in response to the same concentration of Noxa BH3 peptide when compared with MZ-294 cells (Fig 2f) indicating that A172 cells are more sensitive to apoptosis induction as a result of Mcl-1 inhibition than MZ-294 cells.

### R-roscovitine and TRAIL synergise to induce apoptosis in MZ-294 cells

**Fig. 3:**
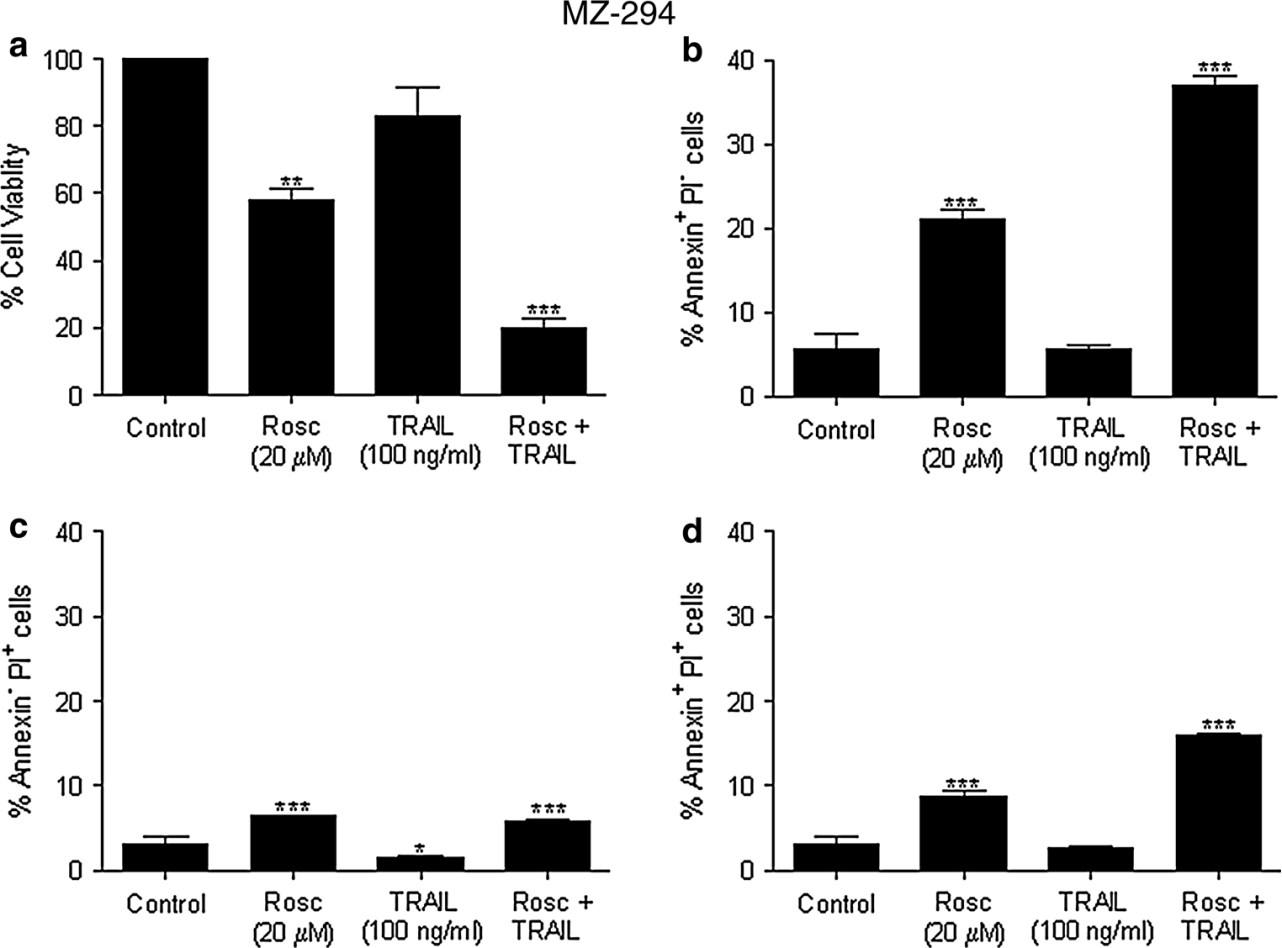
R-roscovitine and TRAIL synergise to induce apoptosis in MZ-294 cells. MZ-294 cells were treated with R-roscovitine (20 μM), TRAIL (100 ng/ml) and R-roscovitine (20 μM) + TRAIL (100 ng/ml) for 48 h. **a** Cell viability following treatment was assessed by MTT assay. The percentage of Annexin^+^PI^−^ **b**, Annexin^−^PI^+^ **c** and Annexin^+^PI^+^ **d** cells following treatment was determined using flow cytometry. Data are expressed as mean ± SEM, **p < 0.01; ***p < 0.001 versus control; representative of three independent experiments

Having established a role for Mcl-1 in the resistance of our GBM cell lines to chemotherapy-induced apoptosis, we investigated if R-roscovitine, given its capacity to decrease Mcl-1, and TRAIL could synergise to induce apoptosis in the treatment-resistant MZ-294 cell line. The cells were treated with either R-roscovitine or TRAIL or a combination of the two for 48 h at concentrations previously used in the literature [34]. There was a significant decrease in the viability of MZ-294 cells (Fig 3a) following treatment with R-roscovitine + TRAIL compared with either treatment alone. This was coupled with a significant increase in the percentage of cells undergoing apoptosis as determined by propidium iodide and Annexin V staining (Fig 3b, c, d) using flow cytometry. A greater percentage of cells were Annexin V-positive and PI-negative (36.9 %; Fig 3b) than were Annexin V-negative and PI-positive (5.8 %; Fig 3c), indicating that the majority of dying cells were undergoing apoptosis [35]. Some cells labelled positive for both Annexin V and PI (15.9 %; Fig 3d) indicating that some cells were also undergoing necrosis, secondary to early apoptosis.

### R-roscovitine + TRAIL treatment induces apoptosis in a 3D tumour model

**Fig. 4:**
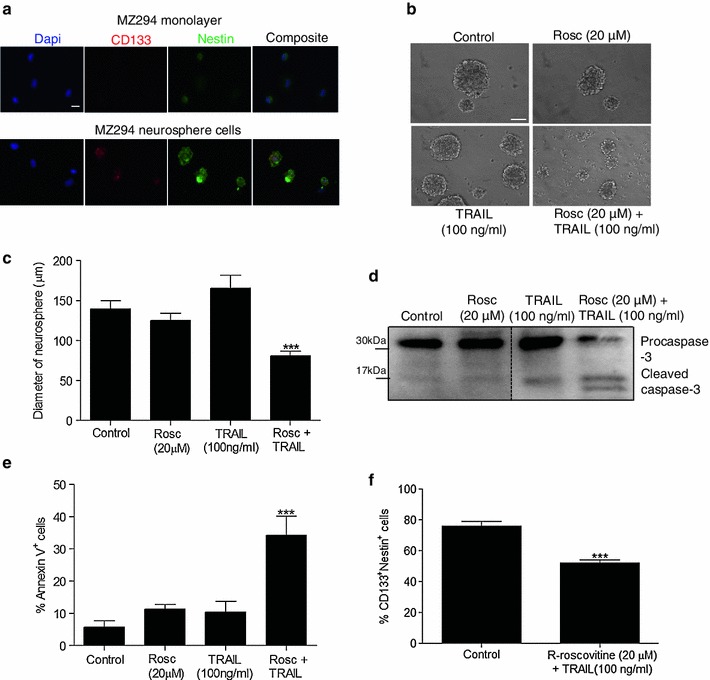
Treatment with R-roscovitine + TRAIL induces apoptosis in a 3D tumour model. MZ-294 neurospheres were generated from MZ-294 monolayer cells cultured in serum-free neurosphere-forming medium. **a** MZ-294 monolayer cells and MZ-294 neurospheres were dissociated and cytospun onto slides and then stained with the stem cell markers CD133, nestin and the nuclear marker Dapi (*scale bar* = 20 μM). **b** MZ-294 neurospheres were treated with R-roscovitine (20 μM), TRAIL (100 ng/ml), R-roscovitine (20 μM) + TRAIL (100 ng/ml) for 48 h. Brightfield images of the neurospheres were taken post treatment (*scale bar* = 50 μM). **c** The diameters of the neurospheres were measured following treatment with R-roscovitine (20 μM), TRAIL (100 ng/ml), R-roscovitine (20 μM) + TRAIL (100 ng/ml) for 48 h using an Eclipse TE 300 inverted microscope. Data are expressed as mean ± SEM, ***p < 0.001; *n* = 40–50 neurospheres. **d** Following treatment, neurospheres were harvested for western blot analysis of the expression of pro-apoptotic proteins procaspase-3 and cleaved caspase-3. **e** The percentage of apoptotic neurosphere cells was determined by Annexin staining using flow cytometry following treatment. Data are expressed as mean ± SEM, ***p < 0.001; data are from three independent experiments. **f** The percentage of neurosphere cells expressing CD133 and Nestin was assessed following treatment with R-roscovitine (20 μM) + TRAIL (100 ng/ml) for 48 h

Neurospheres were generated from MZ-294 cells to form a 3D tumour model. Such models more closely represent the cell–cell interactions and the limited O_2_ and nutrient availability observed in a tumour in situ [36]. Thus we next explored if the combined treatment strategy could also target cells in our 3D GBM tumour model. Dissociated neurospheres generated from MZ-294 cells were stained for CD133 and nestin and were shown to preferentially express these stem cell markers (Fig 4a) indicating that the cells in the 3D tumour model display more undifferentiated properties compared to the MZ-294 monolayer. The neurospheres were allowed to form for 4 d and then treated with R-roscovitine, TRAIL and R-roscovitine + TRAIL for 48 h. Images were taken of the neurospheres (Fig 4b) and the diameters of the neurospheres were measured. Treatment with R-roscovitine + TRAIL significantly decreased the diameters of the neurospheres and their ability to proliferate and grow in size (Fig 4c). Treatment with R-roscovitine + TRAIL also significantly increased the expression of cleaved caspase-3 (Fig 4d), enhanced the percentage of cells in the neurospheres undergoing apoptosis (Fig 4e) and decreased the percentage of cells that were CD133 and nestin positive (Fig 4f).

### R-roscovitine and TRAIL-induced apoptosis in MZ-294 cells is achieved through an Mcl-1/Noxa axis

**Fig. 5:**
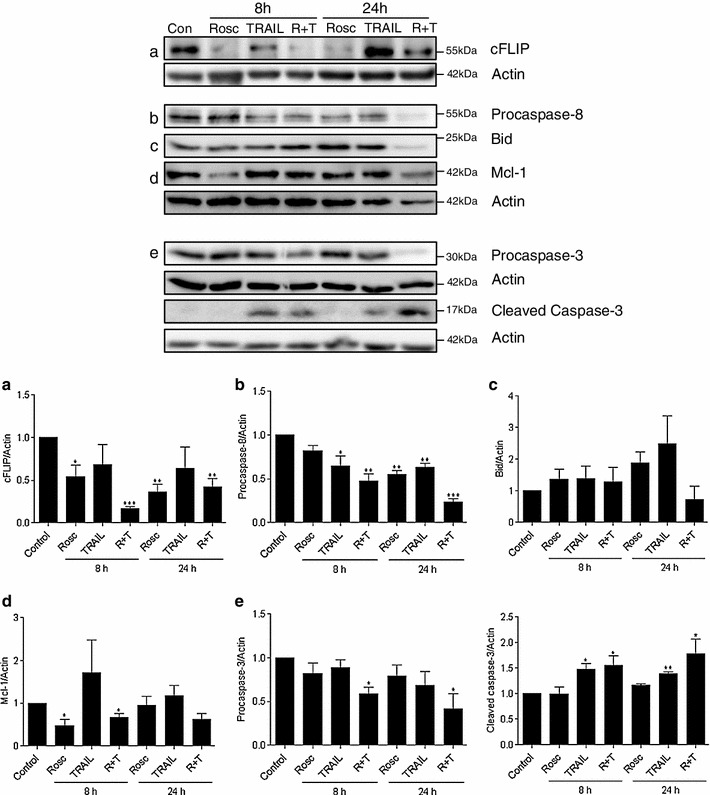
Altered expression of apoptosis-associated proteins in MZ-294 cells following treatment with R-roscovitine + TRAIL. MZ-294 cells were treated with R-roscovitine (20 μM), TRAIL (100 ng/ml), R-roscovitine (20 μM) + TRAIL (100 ng/ml) for 8 and 24 h. *Top panel*: Sample western blot images of the expression of the apoptosis-associated proteins **a** cFLIP, **b** procaspase-8 **c** Bid, **d** the anti-apoptotic protein Mcl-1 and **e** pro and cleaved caspase-3 following treatment. Actin was used as a loading control. *Bottom panel*: Graphs depicting densitometry analysis of the indicated protein levels at 8 h and 24 h following treatment. Data are from three independent experiments. *p < 0.05, **p < 0.01, ***p < 0.001 versus control

**Fig. 6:**
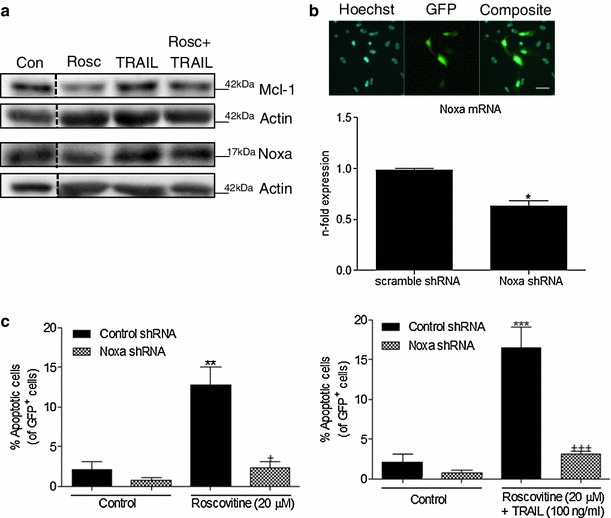
The Mcl-1/Noxa axis plays a critical role in R-roscovitine and TRAIL-induced apoptosis in MZ-294 monolayer cells. **a** MZ-294 cells were treated with R-roscovitine (20 μM), TRAIL (100 ng/ml) and R-roscovitine (20 μM) + TRAIL (100 ng/ml) for 24 h and the expression of Mcl-1 and Noxa was assessed by western blot analysis. Actin was used as a loading control. **b** Images (*scale bar* = 20 μM) depicting transfection efficiency of the GFP-expressing scramble control and Noxa knockdown shRNA in MZ-294 cells and the subsequent knockdown of Noxa after 72 h are shown. **c** MZ-294 cells were transfected with a GFP-expressing Noxa knockdown shRNA and treated with R-roscovitine (20 μM) or R-roscovitine (20 μM) + TRAIL (100 ng/ml) for 48 h. The percentage of apoptotic GFP^+^ cells was determined by Hoechst staining. Data is expressed as mean ± SEM, **p < 0.01; ***p < 0.001 versus control cells; ^+^ *p*<0.05 versus R-roscovitine treated cells; ^+++^ *p* < 0.001 versus R-roscovitine + TRAIL treated cells; data are from three independent experiments

As the combination therapy of TRAIL and R-roscovitine was effective in inducing cell death in both the monolayer and 3D neurospheres of a formerly treatment-resistant GBM cell line, yet R-roscovitine was not capable of altering the expression of the TRAIL receptors DR4 and DR5 (Supplemental Fig 1), we focused our investigation on the downstream cell death signalling pathways elicited by this drug combination. We first determined the expression of cFLIP in monolayer MZ-294 cells following treatment and showed that R-roscovitine has the ability to significantly downregulate the expression of cFLIP at 8 h and 24 h when used alone and when used in combination with TRAIL (Fig 5a; top and bottom panel). As cFLIP is a known inhibitor of death receptor signalling [16], we next assessed the ability of the differing treatment paradigms to activate caspase-8 within the MZ-294 cells (Fig 5b). We observed that treatment with TRAIL alone and in combination with R-roscovitine was sufficient to activate caspase-8 as early as 8 h after treatment and this activation persisted until 24 h (Fig 5b; top and bottom panel). However as significant levels of apoptotic cell death were only evident using the combined treatment strategy (Fig 3), we next focused on signalling events further downstream of caspase-8 activation. Interestingly, while we found no evidence of significant changes in the expression levels of Bid under any of the treatment strategies at the indicated time-points (Fig 5c; top and bottom panel), we did detect activation of caspase-3 as early as 8 h following treatment (Fig 5e; top and bottom panel) and similar to caspase-8, caspase-3 activation persisted until 24 h in both the TRAIL treated and TRAIL and R-roscovitine treated cells (Fig 5e; top and bottom panel). However, as TRAIL treatment alone did not kill the cells (Fig 1) despite such caspase-3 activation, we next examined the levels of Mcl-1 expression, a known target of R-roscovitine (Fig 5d; top and bottom panel). The observed downregulation of Mcl-1 coincided with caspase-8 and caspase-3 activation in the TRAIL and R-roscovitine treated cells while being absent from the TRAIL alone treated cells, indicating that the downregulation of Mcl-1 was a significant event in the cell death observed in the dual treatment strategy. To further investigate the potential contribution of Mcl-1 in our combined treatment paradigm, we examined the expression levels of both Mcl-1 and its pro-apoptotic binding partner Noxa, in MZ-294 monolayer cells following treatment with R-roscovitine, TRAIL and combined R-roscovitine and TRAIL (Fig 6a). In the R-roscovitine-only treated cells, and the dual therapy treated cells we saw downregulation of Mcl-1 protein levels, coupled with a decrease in Noxa expression levels. In the cells treated with TRAIL monotherapy, which did not evoke any cell death (Fig 1a), the expression of both Mcl-1 and Noxa remained similar to control levels. Following transfection with GFP vector expressing a Noxa-targeting shRNA (Fig 6b; top panel), we show significant knockdown of Noxa at the mRNA level (Fig 6b). Counting transfected cells only, pertinently, we saw that knockdown of Noxa significantly attenuated the levels of apoptosis observed following treatment of MZ-294 cells with R-roscovitine alone and R-roscovitine + TRAIL (Fig 6c), demonstrating that Noxa must be present for R-roscovitine and R-roscovitine + TRAIL-induced apoptosis of GBM to proceed.

## Discussion

The significant findings of this study demonstrate that the expression of Mcl-1 in GBM cells plays a critical role in determining their sensitivity to TRAIL-induced apoptosis and that the effective targeting of Mcl-1 in TRAIL resistant GBM cells can establish sensitivity to TRAIL, both within the bulk cell population and crucially within the 3D tumour model. We also highlight that two separate pathways are activated during the apoptotic death of GBM cells treated with the combined chemotherapeutic agents, TRAIL and the Mcl-1-targeting, cyclin-dependent kinase inhibitor, R-roscovitine, one which leads to caspase-8 and caspase-3 activation and a second pathway, involving a Mcl-1:Noxa axis.

The pivotal role Mcl-1 plays in protecting cancer cells from apoptosis is well documented [37]. In GBM cells in particular, the downregulation of Mcl-1 using miRNA has been shown to significantly decrease cell proliferation and increase apoptosis [38]. Additionally, targeting of Mcl-1 using shRNA has been demonstrated to sensitize glioma stem cells to the small molecule inhibitor, ABT-737 [39]. In this study we have found that the downregulation of Mcl-1 is also an effective method to establish TRAIL-induced apoptosis in TRAIL resistant GBM cells. To our knowledge this is the first time that Mcl-1 levels and TRAIL sensitivity have been shown to be inversely correlated in GBM. Our observations emphasize the necessity to investigate Mcl-1 expression levels prior to the therapeutic utilization of TRAIL in GBM patients, especially when one considers the high levels of Mcl-1 expression reported in tumours isolated from such patients [4].

TRAIL has displayed excellent potential as a novel therapeutic agent in the treatment of GBM [19]. It is now well documented that while some glioma cell lines are sensitive to the apoptotic effects of TRAIL, the majority of glioma cell lines and patient samples are resistant [19, 22, 40]; therefore, combinatorial approaches with conventional chemotherapies or novel targeted therapies may be required to ensure the therapeutic potential of TRAIL in GBM treatment is fully exploited [19]. Accordingly cisplatin [41], the pharmacological targeting of Bcl-2 and Bcl-x [22], proteasome inhibitors [40, 42, 43] and histone deacetylase inhibitors [44] have all been proven to be effective at establishing TRAIL sensitivity within GBM. In this study, we demonstrated that strategies aimed at downregulating Mcl-1 expression, such as shRNA or the cyclin-dependent kinase inhibitor, R-roscovitine, have the potential to establish TRAIL-induced killing in GBM. R-roscovitine downregulates Mcl-1 expression through its ability to induce rapid dephosphorylation of RNA polymerase II [45]. Given that R-roscovitine can cross the blood brain barrier [46], this approach to silencing Mcl-1 expression levels may be more clinically feasible than using shRNA technology to enhance TRAIL-induced apoptotic effects in vivo. R-roscovitine-induced inhibitory effects on cyclin-dependent kinases and attenuating effects on Mcl-1 expression levels have been associated with the induction of secondary necrosis in immune cells [47, 48]. While not a direct focus of our study, secondary necrosis may also contribute to the cell death observed. However, the majority of cells were Annexin V-positive and PI-negative suggesting that the preferred pathway in our model system is apoptosis. Our observation that combined R-roscovitine and TRAIL treatment also successfully induced apoptosis in the 3D tumour model, which more accurately represents the tumour environment in vivo, further enhances the potential of this combination as a relevant therapeutic in the treatment of GBM.

In this study, we demonstrate that R-roscovitine-induced sensitisation of MZ-294 cells to TRAIL is not dependent on upregulation of the TRAIL death receptors, DR4 and DR5. The inability of R-roscovitine to alter the expression of DR4 and DR5 was previously reported in a study by Kim et al. [34] and thus our investigations focused on the downstream signalling pathways elicited by the combination therapy of R-roscovitine and TRAIL in our TRAIL resistant GBM cells. We observed the potential contribution from two parallel cell death pathways.

We initially demonstrated that R-roscovitine decreased the expression of the protein, cFLIP, in MZ-294 cells when used alone or in combination with TRAIL. Subsequently, we saw evidence of caspase-8 activation in both the TRAIL alone and combined treatment paradigms, indicating that R-roscovitine-mediated cFLIP downregulation alone was insufficient to trigger caspase-8 activation and that a TRAIL signalling pathway was critical to facilitate caspase-8 activation. Greater levels of caspase-8 activation were achieved using the dual treatments as TRAIL signalling occurred concomitantly with cFLIP downregulation. Previous studies in multiple cancer types, using various methods to downregulate cFLIP expression, have demonstrated similar effects on the enhancement of TRAIL signalling, for e.g., cycloheximide in renal cancer cells [49], histone deacetylase inhibitors in glioma cells [44] and R-roscovitine in breast cancer cells [50]. As Bid is usually described as the main link between the extrinsic and the intrinsic apoptotic pathways in type II cells [51], we next examined the conversion of Bid to tBid and interestingly only observed the appearance of tBid at approximately 72 h post treatment (data not shown). Coinciding with procaspase-8 activation, however, was the activation of procaspase-3 in the TRAIL alone and combined treatment regimens. In the absence of tBid, such cleavage and activation of procaspase-3 may be the result of direct processing by active caspase-8 [52]. Similar to caspase-8 activation, greater levels of active caspase-3 were achieved using the dual treatment strategy. We detected cleaved caspase-3 in the TRAIL alone treated cells, which did not die. Such sub-lethal caspase-3 signalling has previously been reported by Aldridge and colleagues in their models of TRAIL-induced apoptosis [53]. It is interesting to speculate that this sub-lethal level of caspase-3 activation could perhaps be promoting growth rather than death, a role that has previously been suggested for caspase-3 in tumour cells [54]. This is the first time such caspase-3-type activity has been reported for glioblastoma cells and warrants further investigation.

Concurrently, we investigated the role of Mcl-1, a known target of R-roscovitine, in our treatment paradigm. Initially, we observed Mcl-1 downregulation following R-roscovitine treatment; however, this was insufficient to induce a significant level of apoptosis due in part to the lack of caspase-3 activation in these cells. Treatment of the cells with TRAIL alone had little effect on Mcl-1 expression levels, emphasising the requirement for Mcl-1 downregulation in the execution of apoptosis, even in the presence of caspase-3 activation. Following the combined treatment of both R-roscovitine and TRAIL, we observed targeting of Mcl-1, activation of caspase-3 and pertinently, significant levels of cell death were achieved.

Examination of the expression levels of Mcl-1 and Noxa in all of the treatment strategies suggested to us that the interplay between Mcl-1 and Noxa levels played a role in attaining the observed levels of cell death. Previous reports have highlighted the ratio between these two proteins is essential in the sensitization of melanoma cells to ABT-737 [55] and in the sensitization of glioma cells by bortezomib to vorinostat-induced apoptosis [56]. Zhang et al.’s findings add further weight to our hypothesis as their study in HeLa cells highlights that Noxa functions as a constitutive inhibitor of Mcl-1, which remains associated with Mcl-1 even during TRAIL treatment. Upon knockdown of Noxa however, there is a significant decrease in the levels of TRAIL-induced apoptosis as such knockdown results in an effective increase in the levels of free/active Mcl-1 [25]. To verify a role for Noxa in our R-roscovitine + TRAIL treatment strategy, we silenced Noxa protein expression by shRNA targeting and highlighted that this restored resistance to the combined treatment regime in the MZ-294 cells. Such findings are particularly pertinent in light of studies which demonstrate that overexpression of the anti-apoptotic proteins, Bcl-2 or Bcl-xL, neither of which are capable of binding Noxa, had no effect on subsequent combined R-roscovitine and TRAIL treatment in glioma cells [34].

The exact series of events in the final execution of apoptosis by these two parallel death pathways has yet to be fully elucidated. It is interesting to hypothesise that as a result of Mcl-1 downregulation following R-roscovitine and TRAIL treatment, Noxa is capable of displacing the activator BH3-only protein, Bim, and the pro-apoptotic protein, Bak, from Mcl-1:Bim and Mcl-1:Bak complexes respectively [55, 57], thereby triggering mitochondrial dysfunction, caspase activation and ultimately apoptosis [55, 57]. Feeding into this pathway is the direct activation of caspases-8 and -3, which alone do not appear to be sufficient to trigger significant levels of cell death. These active caspases may also contribute to the observed downregulation of Mcl-1 expression following R-roscovitine and TRAIL treatment since both have been shown to mediate cleavage of Mcl-1 and loss of Mcl-1 expression [58], thereby freeing further Bim or Bak. Our observation that the silencing of Noxa protein expression also resulted in resistance to the limited levels of cell death induced by R-roscovitine treatment alone adds further weight to our argument that TRAIL-mediated activation of caspases-8 and -3 makes an important contribution to the significant levels of cell death we observed using the dual treatment strategy.

Taken together, this study provides convincing evidence that a combinational therapeutic treatment regime that modulates Mcl-1 expression levels may be an efficacious approach to sensitize GBM to the apoptosis-inducing effects of TRAIL. Undoubtedly, R-roscovitine in combination with TRAIL presents a promising novel strategy to trigger cell death pathways in glioblastoma and warrants further investigation.

## Electronic supplementary material

Below is the link to the electronic supplementary material.

Supplementary material 1 (PDF 129 kb)

## Abbreviations

Bcl: B cell lymphoma
Bid: BH3 interacting-domain death agonist
BH: Bcl-2 homology
GBM: glioblastoma
GSC: GBM stem cells
Mcl-1: myeloid cell leukaemia-1
Rosc: R-roscovitine
tBid: truncated BID
TRAIL: tumour necrosis factor-related apoptosis-inducing ligand
