# Supplementary material for: Modulation of Mcl-1 sensitizes glioblastoma to TRAIL-induced apoptosis
Source: Apoptosis. 2013 Nov 9;19(4):629–42. doi: 10.1007/s10495-013-0935-2 (PMC3938842; doi:10.1007/s10495-013-0935-2)
Supplement: Supplementary file 1 — Supplementary material 1 (PDF 129 kb) [file 10495_2013_935_MOESM1_ESM.pdf]

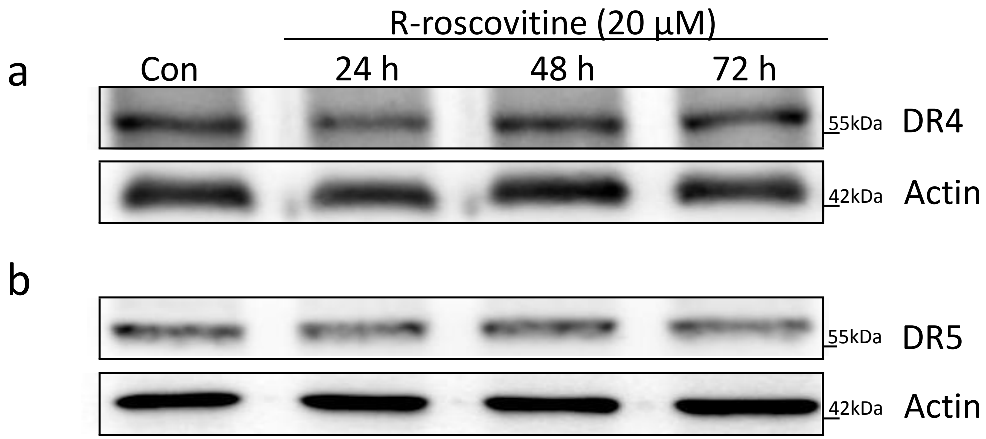

Fig 1

Expression of DR4 and DR5 is not significantly altered in MZ-294 cells following R-roscovitine treatment. MZ-294 cells were treated with R-roscovitine for 24, 48 and 72 hours. The expression of (a) DR4 and (b) DR5 was assessed by western blotting following treatment. Actin was used as a loading control
